# Co-formulant in a commercial fungicide product causes lethal and sub-lethal effects in bumble bees

**DOI:** 10.1038/s41598-021-00919-x

**Published:** 2021-11-05

**Authors:** Edward A. Straw, Mark J. F. Brown

**Affiliations:** Centre for Ecology, Evolution & Behaviour, Department of Biological Sciences, School of Life Sciences and the Environment, Royal Holloway University of London, Egham, TW20 0EX UK

**Keywords:** Ecology, Agroecology

## Abstract

Pollinators, particularly wild bees, are suffering declines across the globe, and pesticides are thought to be drivers of these declines. Research into, and regulation of pesticides has focused on the active ingredients, and their impact on bee health. In contrast, the additional components in pesticide formulations have been overlooked as potential threats. By testing an acute oral dose of the fungicide product Amistar, and equivalent doses of each individual co-formulant, we were able to measure the toxicity of the formulation and identify the ingredient responsible. We found that a co-formulant, alcohol ethoxylates, caused a range of damage to bumble bee health. Exposure to alcohol ethoxylates caused 30% mortality and a range of sublethal effects. Alcohol ethoxylates treated bees consumed half as much sucrose as negative control bees over the course of the experiment and lost weight. Alcohol ethoxylates treated bees had significant melanisation of their midguts, evidence of gut damage. We suggest that this gut damage explains the reduction in appetite, weight loss and mortality, with bees dying from energy depletion. Our results demonstrate that sublethal impacts of pesticide formulations need to be considered during regulatory consideration, and that co-formulants can be more toxic than active ingredients.

## Introduction

Pollination by bees is an essential ecosystem service^1^. However, wild bees are undergoing declines across the globe, with 37% of analysed European bee species (those with sufficient data) suffering population declines^2^. These declines have been linked, in part, to pesticides^3–5^. Pesticides are applied to crops in formulations, which are mixtures of the active ingredient and co-formulants, with the latter being added to aid the efficiency of the active ingredient^6^. The majority of research and regulatory focus is on the active ingredient, not the formulation as a whole or the co-formulants^7–9^. However, the toxicological effects of co-formulants have been consistently underestimated for bees and other non-target organisms, including humans^7–10^. In agricultural environments, bees are exposed to co-formulants through pesticide formulations, and this exposure is likely to be highest for pesticide classes, like fungicides, which are considered to be bee-safe, because they lack mitigation measures to protect bees from exposure.

Fungicides are very widely used plant protection products (PPP’s), with almost half a million metric tonnes applied globally in 2014^11^, and azoxystrobin is one of the most commonly used fungicide active ingredients. While systematic global data are lacking, in 1999 azoxystrobin products were the biggest selling fungicides globally with $415 million of sales^12^. Azoxystrobin was developed by Syngenta^13^, and was the first fungicide of the strobilurin group brought to market^14^. Azoxystrobin’s mode of action is to inhibit fungal mitochondrial respiration as a Quinone Outside Inhibitor^14^. Syngenta’s flagship formulation was Amistar®, although it has now moved out of patent and 66 different azoxystrobin products are available in the United Kingdom (UK) alone^15^. Amistar® is the representative formulation for azoxystrobin in the European Union (EU) (European Food Safety Authority (EFSA)^16^). EU regulators classed azoxystrobin and Amistar® as of ‘low toxicity to bees’ based on lower tier testing which exclusively uses mortality as a measurement of toxicity. Because of the ‘low toxicity to bees’ categorisation, no mitigation measures are required to reduce exposure of bees, and Amistar® can be applied to bee-attractive flowering crops like strawberries while bees are actively foraging on them (Amistar® Label). As such, exposure of bees to Amistar® is very high, with residue monitoring studies consistently finding high levels of azoxystrobin in bee matrices^17,18^. While these studies only measure the residue levels of the active ingredient, and not the residue levels of the co-formulants, it is likely they would be proportionate, meaning that exposure of bees to Amistar® co-formulants will be commensurately high.

There is a range of co-formulant types, including surfactants that reduce surface tension and help the active ingredient penetrate the leaf, solvents that help dissolve the active ingredient in the solution, and emulsifiers that keep the formulation consistent and uniformly mixed^7^. Individual co-formulants are not submitted to the same suite of regulatory testing as active ingredients are, and they are only tested on bees as part of formulations^19^. We have a very poor understanding of the exposure of bees to co-formulants, with only three studies measuring residues in pollen, nectar or wax^20–23^, finding residues as high as 1051 ± 2897 ppb in wax for the surfactant co-formulant nonylphenol ethoxylates. There are relatively few studies that explicitly test the impacts of co-formulants on bees. The solvents N-methyl-2-pyrrolidone and dimethyl sulfoxide have been tested on honeybees^22–28^, with mortality being seen across a range of doses.

Amistar® has three listed co-formulants (Amistar® Material Safety Data Sheet) (see Table 1). Of these, C16-18 alcohols ethoxylated (CAS-no 68439-49-6), which are part of the chemical group alcohol ethoxylates, constitute 10–20% of the formulation and are of most interest. Alcohol ethoxylates are used as surfactants and emulsifiers, serving both to help the active ingredient azoxystrobin penetrate into crops and to stabilise the product^29^. Alcohol ethoxylates are currently being used to replace alkylphenol ethoxylates as surfactant co-formulants because they can synergise with the active ingredient to a greater extent^29^.

**Table 1:** Doses of chemicals used in each treatment group.

Treatment Name	Substance(s)	Dose (µg) per bee
Negative control	Water	0.0
Positive control	Dimethoate	4
Alcohol ethoxylates	C16-18 alcohols, ethoxylated	160
Naphthalenesulfonic acid	Naphthalenesulfonic acid, dime- thyl-, polymer with formaldehyde and methylnaphthalenesulfonic acid, sodium salt acid	80
Benzisothiazol	1,2-benzisothiazol-3(2H)-one	0.4
Co-Formulant mixture	Alcohol ethoxylates, naphthalenesulfonic acid and benzisothiazol	240.4 (sum of all co-formulants)
Amistar	Amistar	*na*

Some kinds of alcohol ethoxylate have been found to synergistically increase mortality when co-applied with a range of insecticide active ingredients in both aphids (*Aphis citricola*) and cockroaches (*Blattella germanica*)^29–31^). This demonstrates that co-formulants are not toxicologically benign and can meaningfully impact a formulation’s toxicology.

We aimed to test the toxicity of the representative formulation for azoxystrobin, Amistar®, and its individual co-formulants to bumble bees. Our design was specifically tailored to identify any potential toxicity of co-formulants or co-formulant mixtures. We used regulatorily sanctioned methods but expanded the range of metrics taken to include sublethal effects^32^. This enabled us to assess whether the limited level of regulatory testing^16^, and its focus on mortality, accurately captures the toxicity of a substance, including any sublethal damage it can cause. It has been proposed that using mortality alone is inappropriate, and that a more fitness-based approach should be adopted^33^. To our knowledge, this is the first study to test each listed co-formulant in a formulation on bees and the first study ever to explicitly test a co-formulant on bumble bees. Based on preliminary work we predicted that the Amistar® treatment would cause significant mortality and gut damage. We hypothesised that bees would compensate for reduced gut function by over-consuming sucrose and that the reduced gut function would cause weight loss.

## Methods

We used Amistar®, a broad-spectrum fungicide, purchased online through Agrigem Ltd (www.agrigem.co.uk), in September 2019. The formulation identifiers are UK MAPP: 18039, Syngenta ID: A12705B. This is the same formulation used in the EFSA bee risk assessment which used Amistar® as a representative formulation for all formulations containing the active ingredient azoxystrobin^16^.

Some of the co-formulants in the formulation are listed in the material safety data sheet that accompanies the bottle, these are listed in Table 1, and in more detail in Supplementary Table S3. This list may not be comprehensive as European Law^34^ explicitly protects whole formulation composition as proprietary knowledge, so we do not know what other ingredients are present. Only co-formulants with specific toxicity classifications need to be listed, which means an unknown number of other co-formulants could be present^34^. From the difference between the appearance of the co-formulant mixture and Amistar® it is likely that there are other ingredients not listed, see Supplementary Fig. S1. Full details on the formulation and co-formulations used, including source and chemical identifiers can be found in the Supplementary Methods. Azoxystrobin is poorly soluble in all the solvents we trialled, and therefore azoxystrobin was not included as an individual treatment, nor in the co-formulant mixture. Regulatory testing of azoxystrobin on honey bees did not detect any lethal effects^16^.

Each co-formulant dose is proportionate to its concentration in the formulation. Calculations, per bee, are based off of 0.8 µL of Amistar® which is equivalent to a 200 µg dose of the active ingredient, azoxystrobin.

Three commercial colonies of the bumble bee *Bombus terrestris audax* were used in the experiments (Agralan, Wiltshire, UK). On arrival, 10 workers per colony were removed and their faeces visually screened for micro-parasites^35^. No infections were detected, and all colonies were thus retained in the experiment.

We used a modified version of an internationally accepted protocol (OECD 247), used by industry and regulators, where deviations from OECD 247 served to increase the richness of data captured. Below we give a brief summary of the method used, and list all deviations from OECD 247, for the base method in full detail see OECD 247.

We housed and weighed worker bees in individual Nicot cages a day in advance of their chemical exposure, and then rank allocated them to treatments based on their weight, with an even distribution of source colonies by treatment. Bees outside the range of 0.1–0.4 g were not used. Number of bees exposed can be seen in Supplementary Table S1. Prior to, and after exposure, bees had access to ad libitum 45% w/w sucrose solution (Thorne, Windsor, UK). Bees were kept at 25 ± 2 °C and 50 ± 10% relative humidity, under red light or darkness.

We exposed bumble bees to the treatments and doses detailed in Table 1. The amount of each co-formulant is equivalent to the amount of that co-formulant in a 0.8 µL dose of Amistar®, which contains 200 µg of azoxystrobin, per bee. In the EFSA honeybee assessment of azoxystrobin, the same dose of 200 µg was used^16^. The co-formulants listed in the Amistar® material safety data sheet are given as ranges, not exact values, so the upper end of the range was used for a conservative risk estimate. Bees who did not consume the droplet were excluded from the entire experiment. While the proportions of bees who did not feed on the droplet did significantly differ between treatments (Fishers Exact Test, *p* =  < 0.0001), with more non-feeders in treatment groups containing alcohol ethoxylates, there is no reason to believe this would impact the results of the study.

Chemical solutions were made fresh on the day of exposure to ensure no degradation occurred before exposure. Amistar®, the co-formulants and the positive control (dimethoate) were diluted in distilled water. The use of dimethoate as a positive control is standard, and reliably achieves > 90% mortality. The negative control solution was distilled water. The chemical solutions, and the negative control, were mixed 50:50 with 33% w/w sucrose to incentivise the bees to drink them. The doses contained within the solutions given to bees are reported in Table 1.

Bees were starved for four hours prior to exposure, and exposed through an 80 µL droplet pipetted into a BD Plastics 5 mL syringe with the tip cut off. This differs from the standard 40 µL used in OECD 247 to allow for low solubility substances to be tested. Consumption was first checked at 2 h, and again at 4 h. The syringe was checked visually to ensure the bees consumed the whole droplet. The syringe of each bee who had consumed the droplet was then filled with 45% w/w sucrose solution, without any pesticide. Mortality was recorded four hours after exposure began, and every 12 h for 120 h. Syringes were weighed every 12 h to track sucrose consumption. Bees were monitored for longer than the 96 h recommended in OECD 247 to ensure all mortality was captured. As soon as they were found dead, any bees who died were weighed then transferred to a 2 mL Eppendorf tube and frozen at − 80 °C, as were any bees who survived the full 120 h.

Bees were removed from the freezer in batches of 8, placed on ice and slowly allowed to defrost before dissection. The abdomen was cut off and was pinned to a black wax plate. The abdomen was cut on one side, and pinned open. 100 µL of 0.8% Ringers solution was pipetted directly onto the gut and another 100 µL onto the wax to the side of the body to prevent desiccation. The honey crop was cut, and the gut transferred to the droplet on the wax. A GXCAM-5 (GT Vision, Suffolk, UK) dissecting scope camera was used to take two images of the midgut at × 10 magnification using supplementary light.

While dissecting the guts of bees exposed to Amistar® during pilot work, gut melanisation was observed, which led to the formal quantification of gut melanisation in this experiment. As a proxy for the level of damage to the gut we used the presence of melanisation (dark brown patches and striations) on the midgut, which are not seen in healthy bees (EA Straw & MJF Brown pers. obs.; also see “Results”). Images of the bee guts were imported into Fiji^36^, converted to 8-bit and then made binary (black or white). The look up table was inverted to highlight any darker areas of the gut. These darker areas were then selected and the analyse particles tool was used to measure their area. This process was repeated twice for each photo, with two photos per gut, and the mean result for each bee was used in analyses. Using a binary colour map caused some areas on the guts of healthy guts to be highlighted, which explains the background noise in all treatments. The scale was set using a photograph of digital callipers at a known value. This allowed the area of gut melanisation to be calculated in mm^2^. This value does not represent the total area of melanisation on the gut, only that visible in the picture of the midgut, which was only one side of the gut. The gut was photographed on the side of the gut facing upwards after dissection, with no efforts made to arrange the gut to highlight damage. The use of Fiji to analyse insect gut lesions is common in the literature (e.g.,^37,38^).

### Statistical analysis

Statistical analyses were carried out in ‘R’ programming software version 3.6.2^39^ All plots were made using ‘ggplot2’ version 3.2.1^40^ and ‘survminer’ version 0.4.6^41^. AIC model simplification was used, with conditional model averaging where no single model had > 95% AIC support. The candidate set of models was chosen by adding the next best supported model until a cumulative > 95% AIC support was reached. ‘MuMIn’ version 1.43.17 was used for model averaging^42^. Parameter estimates and 95% confidence intervals are reported. Confidence intervals not crossing zero indicate a significant effect, so a 95% confidence interval of − 1.00 to 1.00 would not be significant, but a 95% confidence interval of − 2.00 to − 1.00 would be. Model parameters, AIC weights and final models are presented in Supplementary Tables S4–S7. Where a bee lacked a response variable result due to mortality or experimental error it was excluded from that particular analysis, see Supplementary Tables S1 for full numbers by treatment for each analysis. The positive control was excluded from all analyses because the complete mortality at four hours after treatment meant that their other metrics were not meaningful data. Test results for benzisothiazol and naphthalenesulfonic acid are listed, but full test results are only presented in the Supplementary Results. To compare Amistar® against the treatment groups alcohol ethoxylates and co-formulant mixture, the same statistical test was repeated with the data subset of just these treatments and Amistar® as the reference treatment. Results for this are available in the Supplementary Results. Results on the effects of bee weight and colony of origin on the dependent variables are only reported in the main text if significant and are otherwise in the Supplementary Results. Cox proportional hazards models were used to analyse mortality, utilising ‘survival’ version 3.1-8^43^. The full model for mortality used was (Mortality ~ Treatment + Bee Weight + Colony of Origin). Due to zero, or just one instance of mortality, the benzisothiazol and naphthalenesulfonic acid treatment were excluded from mortality analysis. A death at the halfway mark was artificially added to the negative control treatment to allow for meaningful comparison to treatments with mortality. Proportionality of hazards was checked graphically to validate the Cox proportional hazards assumption. Generalised linear models were used to analyse sucrose consumption of bees who survived the full 120 h. Generalised linear models were used to analyse gut melanisation and weight change on all bees. The full models used was (Metric ~ Treatment + Bee Weight + Colony of Origin), with the metric being either Sucrose Consumption, Weight Change or Gut Melanisation. Model assumptions were checked graphically and met.

## Results

### Mortality

All bees in the positive control treatment died within four hours (n = 34), no bees in either the negative control (n = 35) or the benzisothiazol treatment (n = 36) died over the period of 120 h, and only one bee died in the naphthalenesulfonic acid treatment (n = 33).

**Figure 1:**
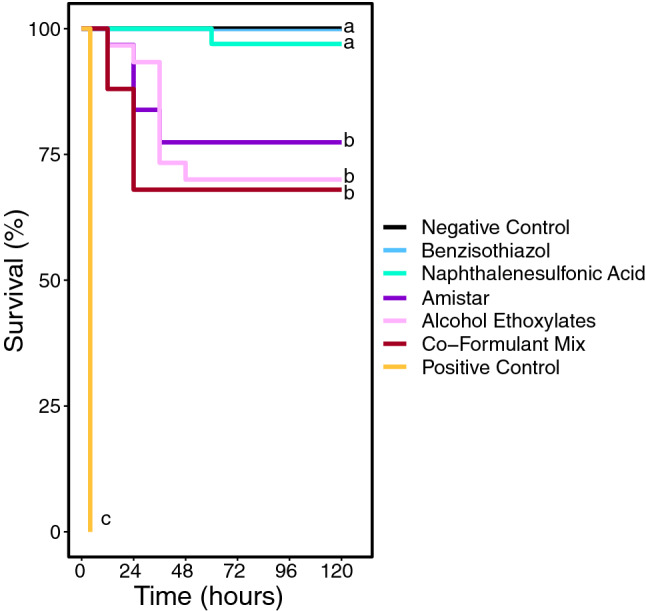
A Kaplan–Meier plot showing survival against time; colour coded by treatment. The negative control and benzisothiazol line is split to allow both to be visible, as both treatments had 0% mortality. Different letters indicate statistically significant differences.

There was significantly higher mortality in all treatments containing alcohol ethoxylates, compared to the negative control. Amistar® (n = 31), co-formulant mixture (n = 25) and alcohol ethoxylates (n = 30) all had significantly higher mortality than the negative control (Cox proportional hazards model: parameter estimate (PE) = 2.16, 95% CI [0.07 to 4.26], (PE) = 2.57, 95% CI [0.65 to 4.83], and PE = 2.28, 95% CI [0.33 to 4.47], respectively). Amistar®, co-formulant mixture and alcohol ethoxylates had 23%, 32% and 30% mortality respectively, while the control (without the artificially added death) experienced 0% mortality (see Fig. 1). Bees whose initial weight was heavier were significantly less likely to die than lighter bees, and bees from one colony were slightly less likely to die also (see Supplementary Results).

### Sucrose consumption

Among the bees who survived the full 120 h there was significantly lower sucrose consumption in all treatments containing alcohol ethoxylates, relative to the negative control.

**Figure 2:**
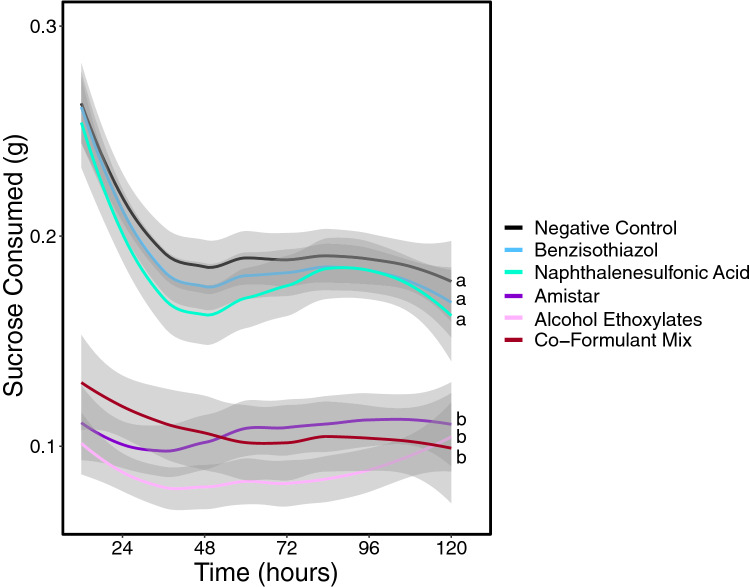
A time series plot showing sucrose consumption over a 120-h period; colour coded by treatment. Average consumption and 95% confidence intervals are shown. Sucrose consumption data collected every 12 h has been LOESS smoothed. Y axis scale refers to sucrose consumption per bee over a 12-h period. Different letters indicate statistically significant differences.

Amistar® (n = 24), co-formulant mixture (n = 17) and alcohol ethoxylates (n = 21) all had significantly lower consumption than the negative control (n = 35) (Generalised linear model: parameter estimate (PE) = − 0.88, 95% CI [− 1.06 to − 0.69], (PE) = − 0.98, 95% CI [− 1.19 to − 0.76], and PE = − 1.06, 95% CI [− 1.26 to − 0.86], respectively). Amistar®, co-formulant mixture and alcohol ethoxylates treated bees consumed an average of 1.086 g, 1.081 g and 0.910 g of sucrose respectively, compared to the 1.973 g in the negative control (see Fig. 2). The difference in sucrose consumption between the Amistar® treatment and the co-formulant mixture and alcohol ethoxylates treatments is not statistically significant. Bees in neither benzisothiazol (n = 36) nor naphthalenesulfonic acid (n = 32) had significantly different consumption versus the negative control (see Supplementary Results). Bees whose initial weight was heavier drank significantly more sucrose than lighter bees, and bees from one colony were significantly more likely to drink slightly less (see Supplementary Results).

### Weight change

There was significantly more weight loss in all treatments containing alcohol ethoxylates relative to the negative control.

**Figure 3:**
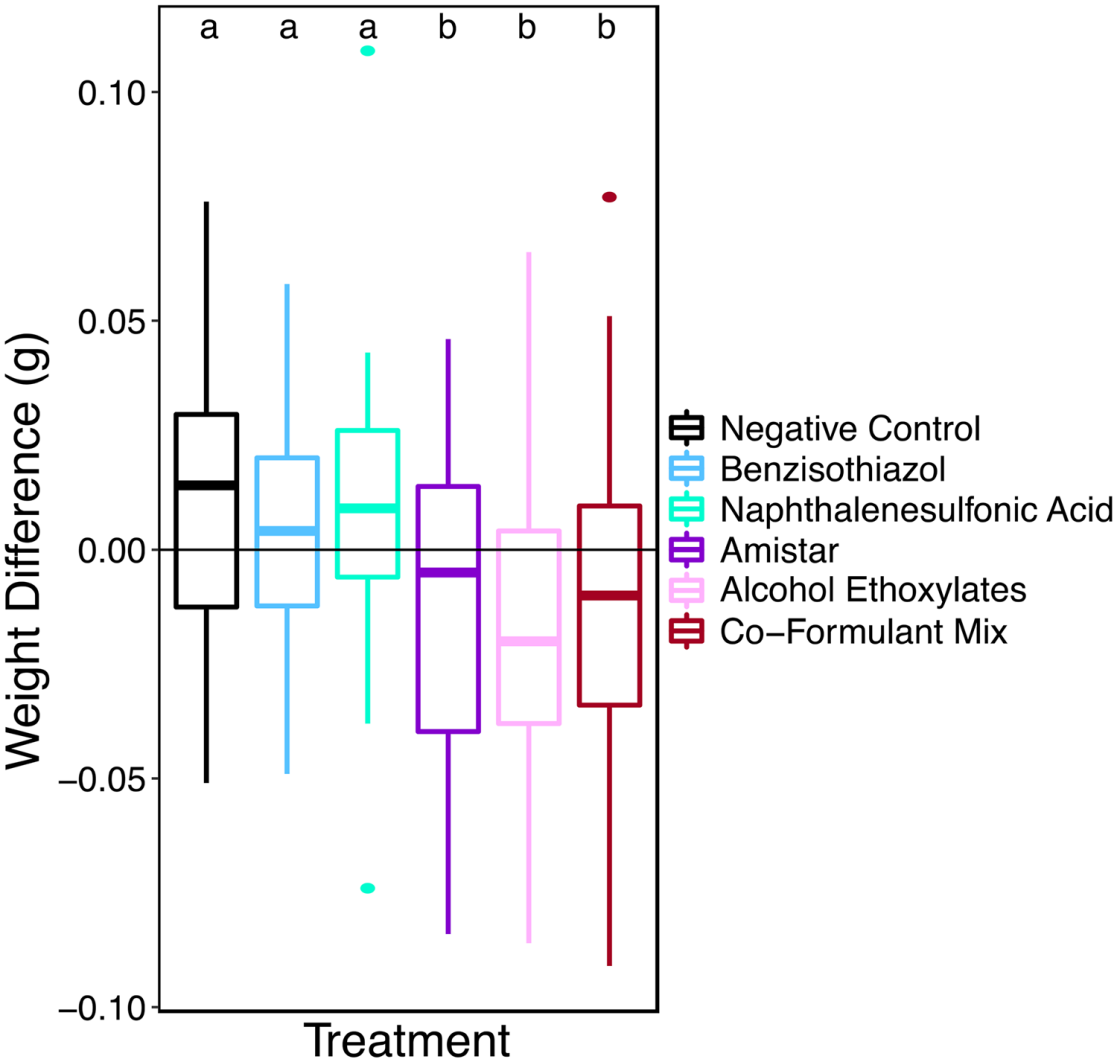
A boxplot showing change in weight over the 120-h period, or until death colour coded by treatment. Boxes represent the Inter-Quartile Range (IQR), with the bold horizontal line the median value. The whiskers represent the furthest datapoint within 1.5 times the IQR and points beyond this are plotted as outliers. Different letters indicate statistically significant differences.

Amistar® (n = 31), co-formulant mixture (n = 25) and alcohol ethoxylates (n = 30) all had a significantly different weight change compared to the negative control (n = 35) (Generalised linear model: parameter estimate (PE) = − 0.02, 95% CI [− 0.03 to − 0.00], (PE) = − 0.03, 95% CI [− 0.04 to − 0.01], and PE = − 0.03, 95% CI [− 0.05 to − 0.02], respectively). Amistar®, co-formulant mixture and alcohol ethoxylates treated bees lost weight over the 120 h, with average losses of 0.010 g, 0.017 g and 0.022 g respectively, in contrast to the negative control where bees gained an average of 0.010 g in the same period (see Fig. 3). The difference in weight change between the Amistar® treatment and the co-formulant mixture and alcohol ethoxylates treatments is not statistically significant. Bees in neither benzisothiazol (n = 36) nor naphthalenesulfonic acid (n = 33) had significantly different consumption versus the negative control (see Supplementary Results). There was no effect of initial weight or colony on weight change (see Supplementary Results).

### Area of gut melanisation

There was significantly more melanisation in all treatments containing alcohol ethoxylates, relative to the negative control.

**Figure 4:**
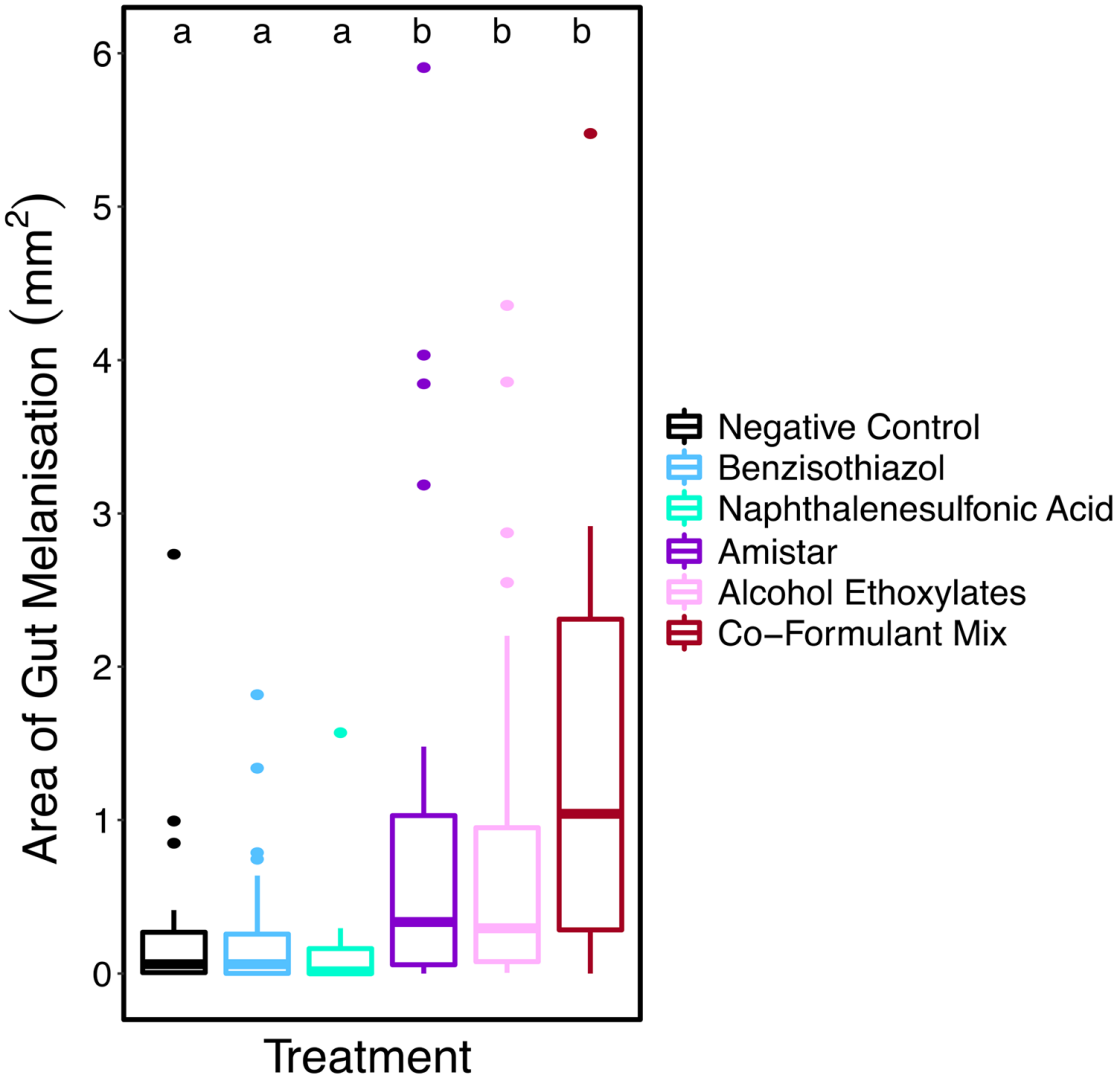
A boxplot showing area of gut melanisation, colour coded by treatment. Boxes represent the IQR, with the bold horizontal line the median value. The whiskers represent the furthest datapoint within 1.5 times the IQR and points beyond this are plotted as outliers. Different letters indicate statistically significant differences.

Amistar® (n = 30), co-formulant mixture (n = 23) and alcohol ethoxylates (n = 29) all had significantly more melanisation than the negative control (n = 35) (Generalised linear model: parameter estimate (PE) = 0.67, 95% CI [0.22 to 1.12], (PE) = 1.13, 95% CI [0.64 to 1.62], and PE = 0.61, 95% CI [0.16 to 1.07], respectively). Amistar®, co-formulant mixture and alcohol ethoxylates treated bees had an average melanised area of 0.925mm^2^, 1.350mm^2^ and 0.850mm^2^ respectively, compared to the 0.230mm^2^ in the negative control (see Fig. 4). The difference in melanised area between the Amistar® treatment and the co-formulant mixture and alcohol ethoxylates treatments is not statistically significant. Bees in neither benzisothiazol (n = 36) nor naphthalenesulfonic acid (n = 33) had significantly different consumption versus the negative control (see Supplementary Results). There was no effect of initial weight or colony on weight change (see Supplementary Results).

## Discussion

Here we show, for the first time, that the toxicity of a pesticide formulation to bees is caused exclusively by a co-formulant (alcohol ethoxylates), rather than the active ingredient. A 0.8 µL acute oral dose of the agricultural fungicide formulation Amistar® caused a range of damage to bees: both lethal, with 23% mortality, and sublethal, with 45% reduced sucrose consumption, 3.8% drop in body weight (whereas the negative control gained 4.8%), and a 302% increase in gut melanisation. For all metrics tested, the Amistar® and alcohol ethoxylates treatments were not statistically different, demonstrating conclusively that the toxicity of the formulation, Amistar®, to bumble bees is driven by the alcohol ethoxylates. These results demonstrate gaps in the regulatory system and highlight the need for a greater research focus on co-formulants.

**Figure 5:**
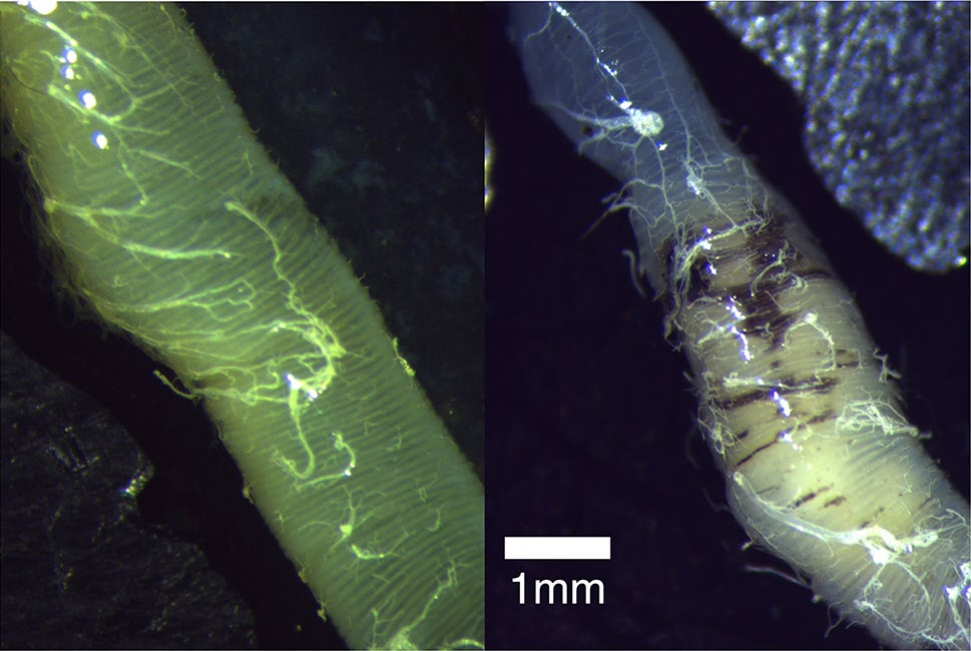
(Left) Bumble bee midgut in the negative control treatment. (Right) Bumble bee midgut in the co-formulant mixture treatment, which contains alcohol ethoxylates. The dark brown patches are areas of melanisation, indicative of damage to the gut. Both bees survived the full 120 h.

The mortality in the Amistar® treatment, and treatments containing alcohol ethoxylates reached 32% at its highest, which is substantial given that bees are likely to have a high level of exposure to Amistar® and alcohol ethoxylates. The mechanism by which the alcohol ethoxylates cause mortality has not been explicitly isolated, but our results suggest two potential, possibly related, causes. We recorded a 302% increase in the melanised area of bee midguts in the alcohol ethoxylates treatment. A similar effect was observed in *Melipona scutellaris* exposed to the pure fungicide active ingredient pyraclostrobin alongside a similar reduction in survival^37^. We suggest that the alcohol ethoxylates are disrupting the structure of the midgut, which the bee immune system is reacting to with melanisation^44^ (see Fig. 5). In parallel with this gut damage, alcohol ethoxylate treatment drove a 54% reduction in sugar consumption, which persisted throughout the experiment. Supplementary Fig. S3 shows a plot comparing sugar consumption against gut melanisation, with increasing gut melanisation correlated to reduced sugar consumption in the Amistar®, co-formulant mixture and alcohol ethoxylates treatments. Consequently, we propose that mortality was driven by energy depletion due to reduced consumption, which in turn may have been driven by damage to the gut.

Likely as a consequence of the reduced consumption of sucrose, bumble bees in the alcohol ethoxylates treatment lost 8.4% of their original weight, in stark contrast to the negative control where bees gained 4.8% over the five-day period. This indicates the alcohol ethoxylate treated bees were expending more energy than they were consuming, and thus exhibiting a negative energy balance. This weight loss, while considerable as a percentage of the bee’s total body mass, is also similar in scale to the weight of the sucrose bees consume in one sitting (EA Straw pers. obs.), for which rigorous data do not exist. As such it is possible that a portion of the weight loss is attributable to the reduced sucrose consumption of the bees, meaning they would have less sucrose in their guts at the time of weighing. Sucrose consumption does not, however, explain the failure of alcohol ethoxylate treated bees to gain weight, which was observed in the control treatment. The weight loss, and lack of weight gain, are concerning because they are likely to indicate a reduction in fat reserves, although this has not been experimentally confirmed. Bee fat reserves are important physiologically, in particular in responding to immune threats^45,46^. Fat reserves allow bees the energetic resources to buffer against challenges, and thus their depletion could expose bees to greater risk from future threats^47^.

The reduced appetite and negative energy balance in alcohol ethoxylates treated bees could have broader effects in the natural environment. Bees pollinate flowers as they forage for nectar and pollen, so a reduction in their appetite could subsequently have effects on ecosystem services. In our experiment, bumble bee appetite was reduced immediately after ingesting a single dose of alcohol ethoxylates or Amistar®. This effect persisted for five days after exposure, indicating a persistent change in consumption behaviour. While nectar-foraging in bumble bees is driven by the needs of the colony^48^, a reduction in appetite would reduce overall colony nectar consumption, and thus the number of foraging trips made for nectar. Fewer visits to flowers for nectar may lead to reduced pollination, which would be detrimental to crop yields and farm profits. Further studies of how the impacts we have found map onto foraging and pollination are clearly needed. Importantly, the reduction in appetite recorded in our experiment is a sublethal effect, which standard lower tier testing would not detect. When Amistar® is tested on bumble bees for the 2025 renewal of azoxystrobin, this sublethal effect will be missed by regulatory testing, despite the impact it may have on the pollination services such testing is designed to protect. We suggest that a simple modification to the regulatory protocol OECD 247 would be to weigh the sucrose syringes at the start and end of the trials to calculate sucrose consumption, which would allow measurement of this sublethal effect with minimal additional workload.

Our results show a slightly, but not significantly, higher level of mortality in the alcohol ethoxylates treatment (30%) than the Amistar® treatment (23%). If this is a real biological difference, one explanation might be that the concentration of alcohol ethoxylates in the Amistar® formulation was lower than that used in the alcohol ethoxylates treatment solution. This is possible because the Amistar® material safety data sheet lists concentrations as a range (10–20% for alcohol ethoxylates), and here we used the upper end of the range. The co-formulant mixture treatment in all metrics was statistically indistinguishable from the alcohol ethoxylates treatment, showing that the toxicity of alcohol ethoxylates is not a result of synergism with other co-formulants.

We believe that the implications of our results are not limited to a laboratory setting and a single species, as other published and unpublished research supports our findings. Semi-field flight cage experiments, where Amistar® was applied to a crop, found effects on full bumble bee colonies (*Bombus terrestris*). Amistar® caused a reduction in average bee weight and a reduction in foraging activity, as our results predict^49,50^. This demonstrates that the effects observed in our laboratory testing scale up to effects at a field realistic level. Additionally, in honeybees (*Apis mellifera*) Amistar® has been found to cause mortality in laboratory experiments at a range of doses^51,52^, demonstrating the mortality effect found in our experiment is not species specific. However, no mortality was seen in trials on the red mason bee *Osmia bicornis* (Hellström and Paxton, unpublished data). Additionally, a similar compound, C11 and lower alcohol ethoxylates, has been found in small scale laboratory testing to cause 100% mortality after contact exposure in honeybees^31^.

To measure the exposure of bees to PPP’s, the EU mandates trials that measure chemical residues in pollen and nectar after crops have been sprayed with either active ingredients or formulations^34^. However, these residue analysis studies only measure active ingredient concentrations, not the co-formulants. As such, we have no systematic data on the exposure of bees to co-formulants^7–9^. This dearth of data means that the exposure of bees to co-formulants is very poorly characterised. To estimate exposure to alcohol ethoxylates, residue data for Amistar®’s active ingredient azoxystrobin could be used as a proxy^18,53^. However, the chemical properties of alcohol ethoxylates, specifically their surfactant action, make it unlikely that they have an equivalent environmental fate to azoxystrobin, so this would not be appropriate.

While we have very little data to quantify bee exposure to alcohol ethoxylates, we know Amistar® can be applied to crops, such as strawberries, during flowering while bees are foraging on them. The Environmental Information Sheet for Amistar® states “[For bees] no risk management is necessary. Amistar® is of low risk to honey bees”^54–56^. In addition, we would note that exposure of bees to alcohol ethoxylates, and related substances, is not exclusively from Amistar®. For example, a cursory search of the Syngenta website^57^ immediately identified alcohol ethoxylates in five other Syngenta products. Worryingly, the chemical group alcohol ethoxylates sit in, alkoxylated alcohols, are also widely used in adjuvants, which are products which can be added to tank mixtures to modify the action of the agrichemical^6^. 89 adjuvant products licenced in the UK contain alkoxylated alcohols as the primary ingredient^15^. To our knowledge, these adjuvants have never been toxicity tested on bees and have no bee exposure mitigation measures in place whatsoever.

To complement measures to promote academic research, moving regulatory research beyond its mortality and active ingredient-centric approach to toxicity testing would better reflect the risks pesticides, as used in the field, pose. For regulatory systems to accurately characterise risk they need to estimate the scale of sublethal effects, regardless of initial mortality results^33^. The results presented here demonstrate that even substances assessed by regulators as ‘bee safe’ can pose a serious hazard to bee health. To reflect potential sublethal differences caused by co-formulation composition, all formulations could undergo a much more rigorous set of lower tier testing or be automatically entered for higher tier testing.

In the face of declining bee populations we advocate that a precautionary approach minimising the exposure of bees to potential stressors, where possible, would be prudent. The current legislation allowing application of PPPs directly onto bees and flowering plants does not align with the emerging evidence that co-formulants, adjuvants, herbicides and fungicides can be hazardous to bees^8,58^. The wealth of untested and undisclosed co-formulants used abundantly in agriculture is a serious and pressing concern for the health of pollinators worldwide.

## Supplementary Information

Supplementary Information.

## Data Availability

Data and code is available at 10.5281/zenodo.5599133.
