# Author Correction: Co-formulant in a commercial fungicide product causes lethal and sub-lethal effects in bumble bees

**DOI:** 10.1038/s41598-022-10434-2

**Published:** 2022-04-13

**Authors:** Edward A. Straw, Mark J. F. Brown

**Affiliations:** Centre for Ecology, Department of Biological Sciences, School for Life Sciences and the Environment, Royal Holloway University of London, Evolution & Behaviour, Egham, TW20 0EX UK

Correction to: *Scientific Reports* 10.1038/s41598-021-00919-x, published online 05 November 2021

The original version of this Article contained an error in Table 1, where the dose for the substance ‘Dimethoate’ was incorrectly given as “0.4 µg”, and now reads “4 µg”.

Additionally, the authors omitted the below Reference, which is listed below as Reference 50.

50. Wintermantel, D. *et al*. Flowering resources modulate the sensitivity of bumblebees to a common fungicide. *Sci. Tot. Environ.* 829, 154450 (2022).

As a result, in the Discussion section,

“Amistar® caused a reduction in average bee weight and a reduction in foraging activity, as our results predict^49^.”

now reads:

“Amistar® caused a reduction in average bee weight and a reduction in foraging activity, as our results predict^49,50^.”

As a result of the changes, the References have been renumbered.

The original Article has been corrected.

