# Supplementary material for: Co-formulant in a commercial fungicide product causes lethal and sub-lethal effects in bumble bees
Source: Sci Rep. 2021 Nov 5;11:21653. doi: 10.1038/s41598-021-00919-x (PMC8571393; doi:10.1038/s41598-021-00919-x)
Supplement: Supplementary file 1 — Supplementary Information. [file 41598_2021_919_MOESM1_ESM.docx]

**Supplementary information:**

**Co-formulant in a commercial fungicide product causes lethal and sub-lethal effects in bumble bees**

*Edward A. Straw^1*^, Mark J. F. Brown^1^*

*Corresponding Author: EdwardAStraw@gmail.com

^1^Centre for Ecology, Evolution & Behaviour, Department of Biological Sciences, School for Life Sciences and the Environment, Royal Holloway University of London, Egham, Surrey, TW20 0EX, UK.

**Supplementary Methods**

Supplementary Table 1. Number of datapoints by treatment for each analysis done.

| Treatment Abbreviation | Mortality *n*= | Sucrose Consumption *n*= | Weight Change *n*= | Area of Melanisation *n*= |
| --- | --- | --- | --- | --- |
| Negative control | 35 | 35 | 35 | 35 |
| Positive control | 34 | 0 | 0 | 0 |
| Alcohol ethoxylates | 30 | 21 | 30 | 29 |
| Naphthalenesulfonic acid | 33 | 32 | 33 | 33 |
| Benzisothiazol | 36 | 36 | 36 | 36 |
| Co-formulant mixture | 25 | 17 | 25 | 23 |
| Amistar | 31 | 24 | 31 | 30 |

All treatment groups started with 35-37 workers, but because some bees did not consume the whole treatment droplet, and some bees died prior to exposure, sample sizes per treatment group changed.

**Statistical analysis**

The negative control treatment was the reference used for comparison of the remaining treatments (Amistar®, co-formulant mixture and alcohol ethoxylates) for mortality testing. Because the negative control experienced no mortality, which causes a failure of the model to converge, we changed the mortality data for a single randomly selected negative control bee who survived the full 120 hours to a death at the halfway mark, 60 hours. This allowed for a meaningful comparison with the remaining treatments while being an even more conservative estimate of the effect size. This manipulation would only serve to reduce the probability of finding a significant result and is an accepted practice in mortality analysis.

Supplementary Table 2. Listed ingredients in Amistar, taken from the material safety data sheet (Amistar Material Safety Data Sheet)

| Substance(s) | Concentration in Pure Formulation (%) |
| --- | --- |
| Azoxystrobin | 20-25% |
| C16-18 alcohols, Ethoxylated | 10-20% |
| Naphthalenesulfonic acid, dime- thyl-, polymer with formaldehyde and methylnaphthalenesulfonic acid, sodium salt acid | 1-10% |
| 1,2-benzisothiazol-3(2H)-one | 0.025-0.05% |

The MSDS for Amistar® includes the information provided in Supplementary Table 2. The upper end of the concentration ranges was used to inform the doses chosen. All doses are proportionate to their concentrations in Amistar® relative to a 200µg dose of the active ingredient azoxystrobin, which is equivalent to 0.8µL of Amistar® pure formulation.

Supplementary Table 3. Full details for formulation, active ingredients and co-formulants used in the experiment. Ministerially Approved Pesticide Product (MAPP).

| Brand Name | Azoxystrobin concentration Pure (g/L) | MAPP | Syngenta ID | Cas No | Producer | Purchased From |
| --- | --- | --- | --- | --- | --- | --- |
| Amistar | 250 | 18039 | A12705B | NA | Syngenta, Cambridge UK | Agrigem.co.uk, Lincoln, UK |
| C16-18 alcohols, Ethoxylated | 0 | NA | NA | 68439-49-6 500-212-8 | Making Cosmetics | Amazon, London UK |
| Naphthalenesulfonic acid | 0 | NA | NA | 9084-06-4 | Sigma Aldrich, Gillingham  UK | Sigma Aldrich, Gillingham  UK |
| 1,2-benzisothiazol-3(2H)-one | 0 | NA | NA | 2634-33-5 220-120-9 613-088-00-6 | Sigma Aldrich, Gillingham  UK | Sigma Aldrich, Gillingham  UK |
| Dimethoate | 0 | NA | NA | 60-51-5 | Sigma Aldrich, Gillingham  UK | Sigma Aldrich, Gillingham  UK |

**
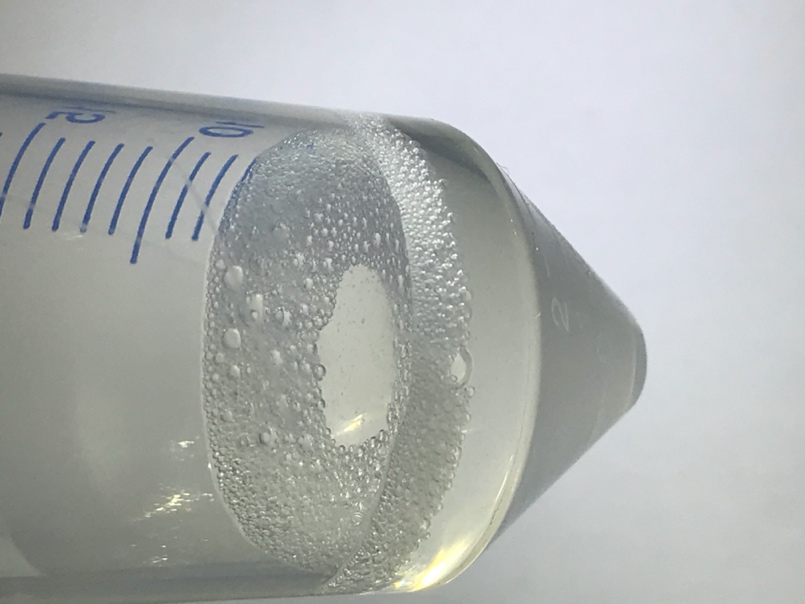

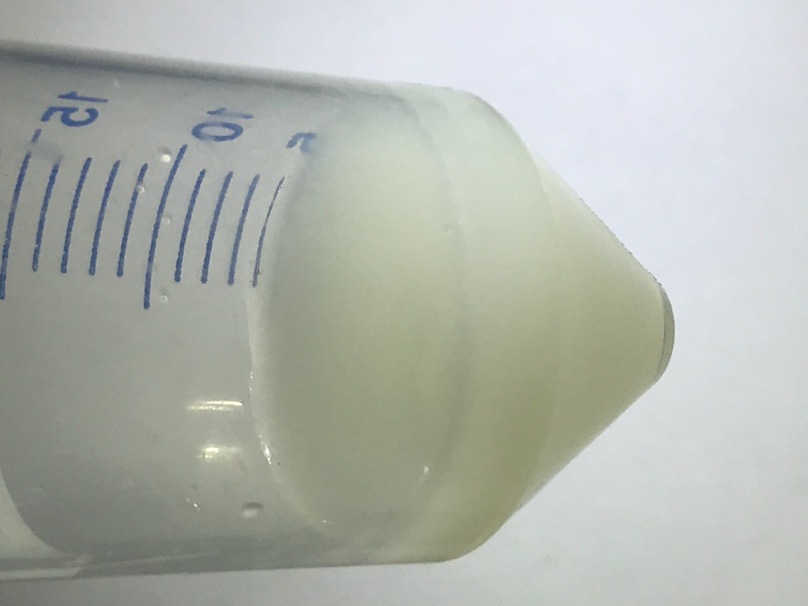
Supplementary Results**

Supplementary Figure 1: (Left) Amistar treatment solution. (Right) co-formulant mixture treatment solution.

The only listed ingredient missing from the co-formulant mixture that is present in the Amistar® is azoxystrobin, which when diluted is not beige or milky. This indicates that there are likely to be additional co-formulants not listed on the material safety data sheet, although it cannot be ruled out that manufacturing process explains the difference. To dissolve azoxystrobin we trialled both acetone and water, with neither being suitable because flocculation occurred once mixed with sucrose.


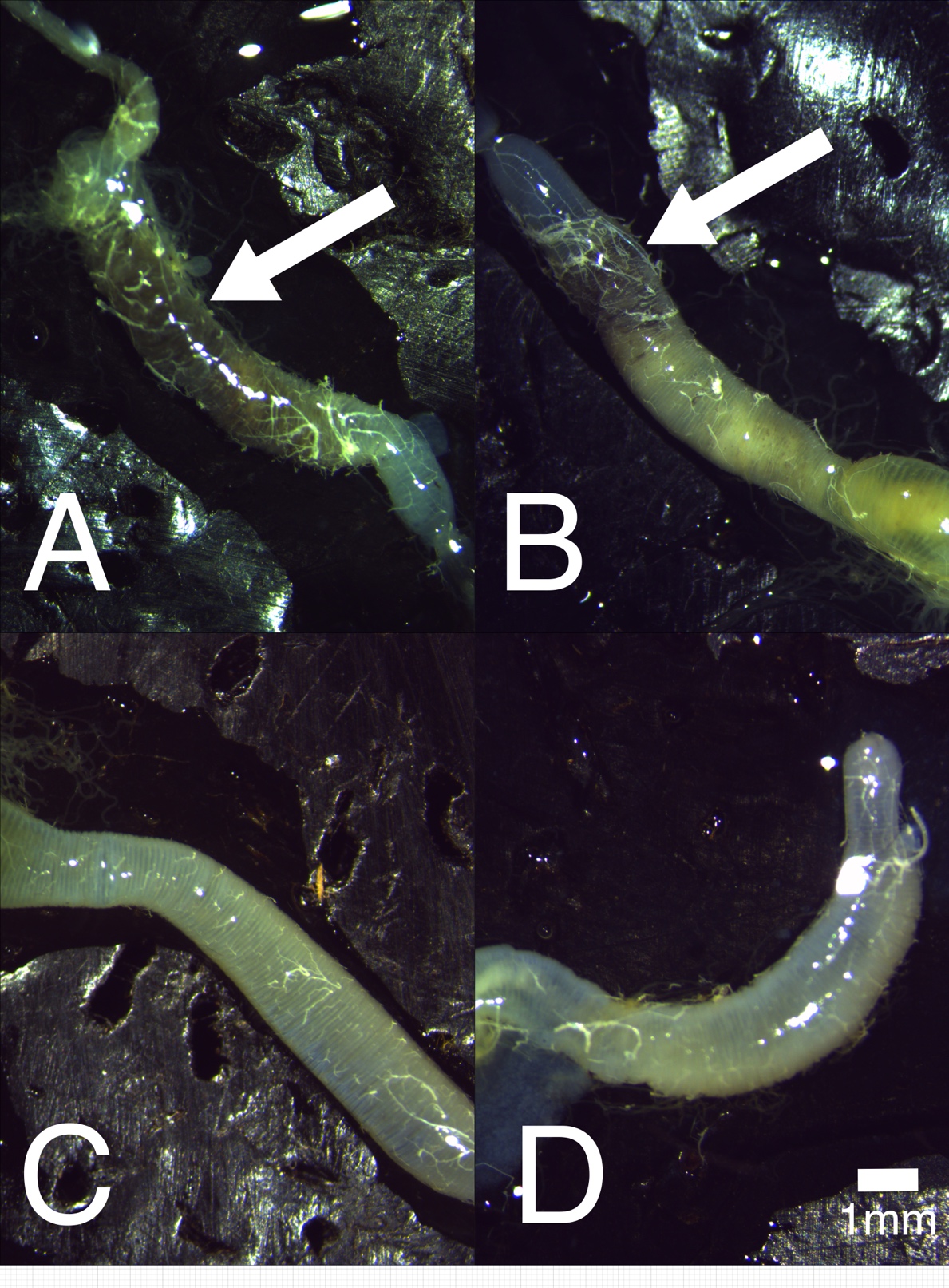
Supplementary Figure 2. Bumble bee foreguts of bees who survived the full 120 hours.

(Top Left-A) Amistar treatment. A large area of the gut is visibly darkened. (Top Right-B) alcohol ethoxylates treatment. A large area of the gut is visibly darkened, and some brown spots are visible. (Bottom left-C) Naphthalenesulfonic acid treatment. No melanisation is visible. (Bottom Right-D) Benzisothiazol treatment. No melanisation is visible. The images in supplementary Figure 2 are sample pictures, and all images are available upon request from the authors.


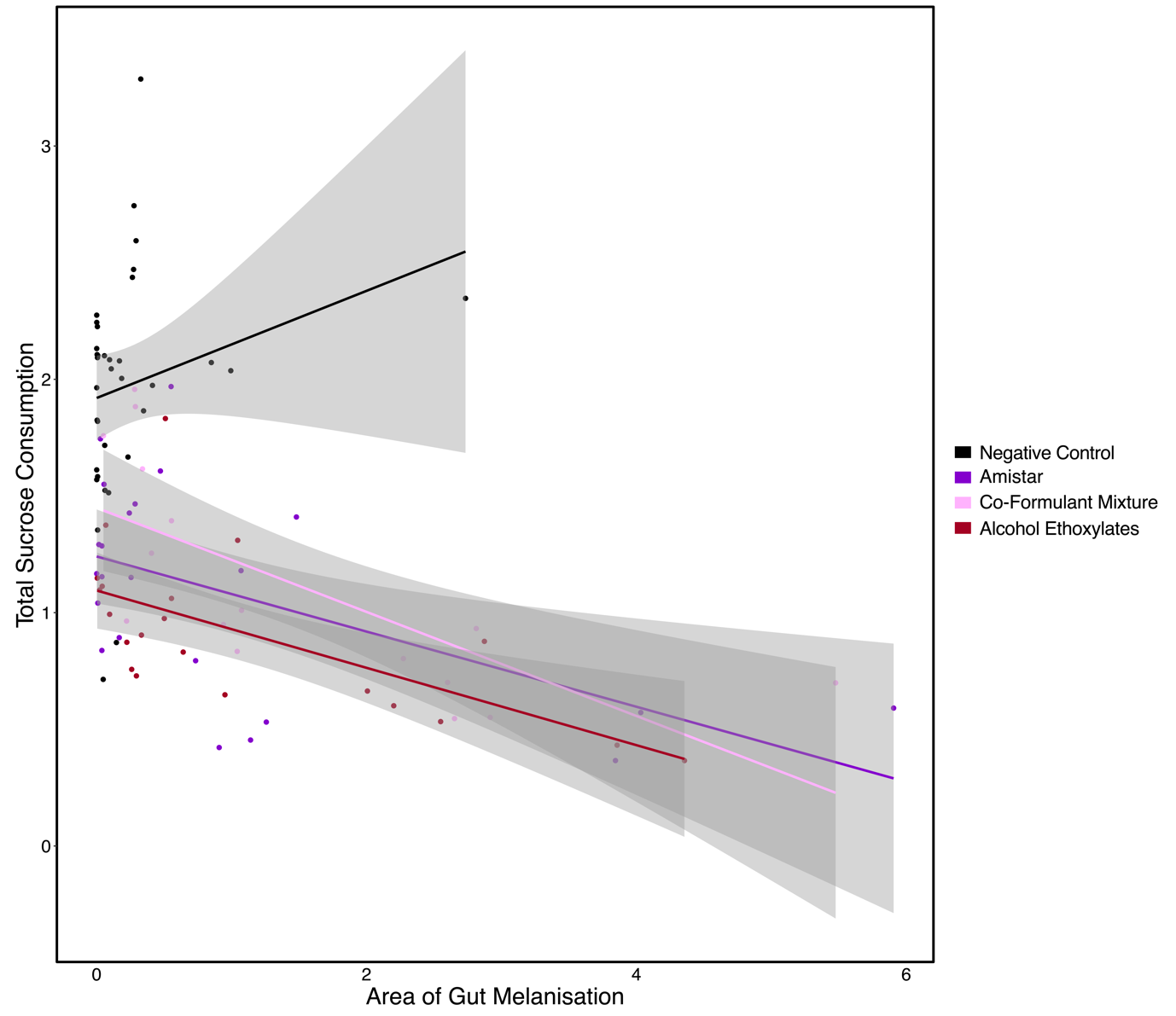
Supplementary Figure 3. Total Sucrose Consumption per bumble bee plotted against area of gut melanisation, with 95% CI. Naphthalenesulfonic acid and benzisothiazol treated bees have been omitted to aid the clarity of the graph. A correlation between increasing area of gut melanisation and reduced sucrose consumption is visible.

**Supplementary Results: Mortality**

Supplementary Tables 4. Mortality: The results of the model selection process for each analysis using the package ‘MuMIn’ (Bartoń 2020). Predictors, AIC, ∆AIC from the Best Model, AIC Weight and whether the model was included in the final parameter estimates are all presented.

| Model Name | Predictors | AIC | ∆AIC from best model | AIC Weight | Included in Final Model Set |
| --- | --- | --- | --- | --- | --- |
| FM | Treatment, Bee Weight, Colony of Origin | 225.1 | 0.00 | 0.352 | Yes |
| M1 | Treatment, Colony of Origin | 225.7 | 0.59 | 0.262 | Yes |
| M2 | Treatment, Bee_Weight | 225.7 | 0.58 | 0.263 | Yes |
| M3 | Treatment | 228.2 | 3.09 | 0.075 | Yes |
| M0a | Colony of Origin | 229.7 | 4.60 | 0.011 | No |
| M0b | Bee Weight | 232.3 | 7.19 | 0.246 | No |
| M0c | Nothing | 233.5 | 9.38 | 0.011 | No |

The slightly lower mortality seen in the Amistar® treatment versus co-formulant mixture and alcohol ethoxylates, is not statistically significant (Cox proportional hazards model: ﻿parameter estimate PE = 0.08, 95% CI [-0.48 to 0.65] and (PE) = 0.04, 95% CI [-0.38 to 0.45], respectively).

There was a small effect of bee weight at the beginning of the experiment on mortality (Cox proportional hazards model: ﻿parameter estimate (PE) = -9.31, 95% CI [-17.89 to -0.74]), with heavier bees less likely to die.

There was a significant effect of colony of origin on mortality for one of two colonies (Generalised linear model: ﻿parameter estimate (PE) = -1.73, 95% CI [-3.22 to -0.24] and PE = -0.41, 95% CI [-1.36 to 0.54]), when compared to an arbitrarily chosen reference colony.

**Supplementary Results: Sucrose Consumption**

Supplementary Tables 5. Sucrose Consumption: The results of the model selection process for each analysis using the package ‘MuMIn’ (Bartoń 2020). Predictors, AIC, ∆AIC from the Best Model, AIC Weight and whether the model was included in the final parameter estimates are all presented.

| Model Name | Predictors | AIC | ∆AIC from best model | AIC Weight | Included in Final Model Set |
| --- | --- | --- | --- | --- | --- |
| FM | Treatment, Bee Weight, Colony of Origin | 150.4 | 0.00 | 1.000 | Yes |
| M1 | Treatment, Colony of Origin | 181.2 | 30.80 | 0.000 | No |
| M2 | Treatment, Bee_Weight | 180.4 | 30.05 | 0.000 | No |
| M3 | Treatment | 202.0 | 51.62 | 0.000 | No |
| M0a | Colony of Origin | 296.7 | 145.35 | 0.000 | No |
| M0b | Bee Weight | 302.4 | 152.00 | 0.000 | No |
| M0c | Nothing | 307.2 | 156.78 | 0.000 | No |

Neither benzisothiazol nor naphthalenesulfonic acid had significantly different consumption versus the control (Generalised linear model: ﻿parameter estimate (PE) = -0.05, 95% CI [-0.22 to 0.12] and PE = -0.15, 95% CI [-0.33 to 0.02], respectively), with an average sucrose consumption of 1.905g and 1.823g of sucrose respectively, compared to the 1.973g in the negative control (see Main Text Figure 2).

The difference in sucrose consumption between the Amistar® treatment and the co-formulant mixture and alcohol ethoxylates treatments is not statistically significant (Generalised linear model: ﻿parameter estimate (PE) = -0.09, 95% CI [-0.34 to 0.15] and PE = -0.21, 95% CI [-0.43 to 0.02], respectively).

There was a significant effect of bee weight on sucrose consumption (Generalised linear model: ﻿parameter estimate (PE) = 3.66, 95% CI [2.44 to 4.86]), with heavier bees drinking more*.*

There was a significant effect of colony of origin on mortality for one of two colonies (Generalised linear model: ﻿parameter estimate (PE) = -0.42, 95% CI [-0.55 to -0.28] and PE = --0.12, 95% CI [-27 to 0.03]), when compared to an arbitrarily chosen reference colony.

**Supplementary Results: Weight Change**

Supplementary Tables 6. Weight Change: The results of the model selection process for each analysis using the package ‘MuMIn’ (Bartoń 2020). Predictors, AIC, ∆AIC from the Best Model, AIC Weight and whether the model was included in the final parameter estimates are all presented.

| Model Name | Predictors | AIC | ∆AIC from best model | AIC Weight | Included in Final Model Set |
| --- | --- | --- | --- | --- | --- |
| FM | Treatment, Bee Weight, Colony of Origin | -760.2 | 2.65 | 0.191 | Yes |
| M1 | Treatment, Colony of Origin | -754.2 | 8.70 | 0.009 | No |
| M2 | Treatment, Bee_Weight | -762.9 | 0.00 | 0.719 | Yes |
| M3 | Treatment | -758.5 | 4.37 | 0.191 | Yes |
| M0a | Colony of Origin | -737.4 | 17.51 | 0.000 | No |
| M0b | Bee Weight | -745.4 | 152.00 | 0.000 | No |
| M0c | Nothing | -741.4 | 21.468 | 0.000 | No |

Neither benzisothazol nor naphthalenesulfonic acid had significantly different weight change versus the negative control (Generalised linear model: ﻿parameter estimate (PE) = -0.01, 95% CI [-0.02 to 0.01] and PE = -0.00, 95% CI [-0.02 to 0.02], respectively), with an average weight gain of 0.005g and 0.010g respectively, compared to the 0.010g gain in the negative control (see main text Figure 3).

The difference in weight change between the Amistar® treatment and the co-formulant mixture and alcohol ethoxylates treatments is not statistically significant (Generalised linear model: ﻿parameter estimate (PE) = -0.01, 95% CI [-0.01 to 0.01] and PE = -0.01, 95% CI [-0.02 to 0.01], respectively).

There was no significant effect of bee weight on weight change (Generalised linear model: ﻿parameter estimate (PE) = -0.11, 95% CI [-0.22 to 0.00]).

There was no significant effect of colony of origin on weight change (Generalised linear model: ﻿parameter estimate (PE) = 0.00, 95% CI [-0.00 to 0.01] and PE = -0.00, 95% CI [-0.01 to 0.01], for either colony compared to an arbitrarily chosen reference colony).

**Supplementary Results: Gut Melanisation**

Supplementary Tables 7. Gut melanisation: The results of the model selection process for each analysis using the package ‘MuMIn’ (Bartoń 2020). Predictors, AIC, ∆AIC from the Best Model, AIC Weight and whether the model was included in the final parameter estimates are all presented.

| Model Name | Predictors | AIC | ∆AIC from best model | AIC Weight | Included in Final Model Set |
| --- | --- | --- | --- | --- | --- |
| FM | Treatment, Bee Weight, Colony of Origin | 509.1 | 3.94 | 0.108 | Yes |
| M1 | Treatment, Colony of Origin | 513.2 | 8.06 | 0.014 | No |
| M2 | Treatment, Bee_Weight | 505.1 | 0.00 | 0.779 | Yes |
| M3 | Treatment | 509.3 | 4.14 | 0.099 | Yes |
| M0a | Colony of Origin | 539.4 | 34.23 | 0.000 | No |
| M0b | Bee Weight | 533.5 | 28.38 | 0.000 | No |
| M0c | Nothing | 536.2 | 31.11 | 0.000 | No |

Neither benzisothiazol nor naphthalenesulfonic acid had significantly different melanised area versus the negative control (Generalised linear model: ﻿parameter estimate (PE) = -0.01, 95% CI [-0.44 to 0.42] and PE = -0.12, 95% CI [-0.56 to 0.32], respectively), with an average melanised area of 0.240mm^2^ and 0.116mm^2^ respectively, compared to the 0.230mm^2^ in the negative control (see main text Figure 4).

The difference in melanised area between the Amistar® treatment and the co-formulant mixture and alcohol ethoxylates treatments is not statistically significant (Generalised linear model: ﻿parameter estimate (PE) = 0.50, 95% CI [-0.45 to 0.76] and PE = -0.04, 95% CI [-0.38 to 0.36], respectively).

There was no significant effect of bee weight on gut melanisation (Generalised linear model: ﻿parameter estimate (PE) = -3.33, 95% CI [-6.18 to 0.19]).

There was no significant effect of colony of origin on weight change (Generalised linear model: ﻿parameter estimate (PE) = -0.04, 95% CI [-0.12 to 0.11] and PE = -0.12, 95% CI [-0.15 to 0.12], for either colony compared to an arbitrarily chosen reference colony).
